# Excessive behaviors in clinical practice—A state of the art article

**DOI:** 10.3402/qhw.v11.30055

**Published:** 2016-02-12

**Authors:** Elisabeth H. Punzi

**Affiliations:** Department of Psychology, University of Gothenburg, Gothenburg, Sweden

**Keywords:** Excessive behaviors, behavioral addictions, assessment, treatment, clinical practice, interaction

## Abstract

This paper concerns difficulties with excessive food intake, sexual activities, romantic relationships, gambling, Internet use, shopping, and exercise—behaviors that might cause considerable suffering. Excessive behaviors are seen as expressions of underlying difficulties that often co-occur with other psychological difficulties, and behaviors may accompany or replace each other. Moreover, they might pass unnoticed in clinical practice. Given the complexity of excessive behaviors, integrated and individualized treatment has been recommended. This paper presents an overview of the terminology concerning excessive behaviors, and the impact of naming is acknowledged. Thereafter, methods for identification and assessment, as well as treatment needs are discussed. Because identification, assessment, and treatment occur in an interaction between client and practitioner, this paper presents a discussion of the need to empower practitioners to identify and assess excessive behaviors and provide an integrated treatment. Moreover, the need to support practitioners’ capacity to handle and tolerate the overwhelming suffering and the negative consequences connected to excessive behaviors is discussed. Qualitative studies are suggested in order to understand the meaning of excessive behaviors, treatment needs, and the interaction between client and practitioner.

During recent decades, difficulties with excessive food intake, sexual activities, romantic relationships, gambling, Internet use, shopping, and exercise have gained attention in research, clinical practice, and popular media (Johnston, Reilly, & Kremer, 2011; Karim & Chaudhri, 2012; MacDonald, 2008; Plant & Plant, 2003; Sussman, Lisha, & Griffiths, 2011; Walther, Morgenstern, & Hanewinkel, 2012). Excessive behaviors seem to be increasing in the general population as well as among clients in mental health care (Gorse & Lejoyeux, 2011; MacDonald, 2008; Reimersma & Sytsma, 2013; Sussman et al., 2011). Excessive behaviors relatively frequently co-occur with substance misuse (Plant, Miller, & Plant, 2005). For example Nökleby (2013) explains that difficulties with food intake are relatively common among clients with substance misuse and Hartman, Ho, Arbour, Hambley, and Lawson (2012) note that excessive sexual activities frequently co-occur with substance misuse. Moreover, interpersonal and affective problems tend to accompany difficulties with gambling (Caputo, 2015), excessive sexual activities (Giugliano, 2003), and food intake (Bromberg, 2001; MacDonald, 2008).

Excessive behaviors might be the reason for clients to seek treatment, but clients might also seek treatment for a variety of reasons without mentioning difficulties with excessive behaviors. Practitioners in mental health care therefore need to pay attention to excessive behaviors, even though the client might not mention excessive behaviors when he or she enters treatment (Freimuth et al., 2008; McKeague, 2014; Power, 2005; Toneatto & Brennan, 2002). Individuals might have difficulties with one or several behaviors. Behaviors and even substances might furthermore replace each other, so that when one excessive behavior is terminated it might be substituted with another behavior or a substance (Freimuth et al., 2008; Goodman, 2008; Shaffer et al., 2004; Sussman et al., 2011). Power (2005) as well as Punzi and Fahlke (2015) note that practitioners seem to be aware that their clients might experience difficulties with excessive behaviors even when treatment is sought for other reasons, and practitioners also seem to be aware that excessive behaviors might accompany as well as replace each other. Nevertheless, excessive behaviors can pass unnoticed in clinical practice and therefore the capacity to identify and approach these behaviors needs to be enhanced (Fernandez-Aranda et al., 2006; Freimuth et al., 2008; Plant & Plant, 2003; Power, 2005; Punzi & Fahlke, 2015; Robbins & Clark, 2015).

In research concerning excessive behaviors, two main perspectives can be traced. On one hand, there are researchers who view these behaviors as separate entities that share common features but nevertheless should be researched and treated as singular conditions. For those researchers, it is important to specify the characteristics and consequences of each behavior separately. Moreover, when methods of identification, assessment, and treatment are investigated, the investigations are specific to the behavior concerned. This is a relevant approach since each specific excessive behavior holds characteristics and consequences that are irrelevant for other behaviors but of importance for the behavior concerned. If excessive eating, for example, was specifically being investigated, it might be relevant to study interventions concerning diets and weight, whereas such interventions do not have prominence if for example excessive shopping was being investigated. On the other hand, there are researchers who view excessive behaviors as varying expressions of the same underlying difficulties (Bromberg, 2001; Giugliano, 2003; Goodman, 2008; Orford, 2001). These researchers view excessive behaviors as replaceable by other behaviors and sometimes also by substances. According to researchers working from this perspective, knowledge and methods concerning one behavior might be used with respect to other behaviors, including excessive intake of substances. The author of this paper is aware that clients might have difficulties with one specific behavior that will not be replaced by others if it is terminated, and considers it important to investigate behaviors as separate entities. Even though excessive behaviors might replace each other it is important to pay attention to differences between the development of the behaviors and the different circumstances in which they are enacted. For example, gambling is traditionally enacted in contexts in which alcohol is available, a prerequisite that increases the risk for co-occurring difficulties with alcohol and gambling (Sussman et al., 2011; Svensson, Romild, Nordenmark, & Månsdotter, 2011). By contrast, excessive exercise is enacted in contexts in which bodily appearance is highly valued, and accordingly it seems to co-occur with difficulties with food intake (Sussman et al., 2011; Warner & Griffiths, 2006). With awareness of the differences between behaviors, this paper concerns excessive behaviors as expressions of underlying difficulties—behaviors that might be replaced by each other and sometimes even by substances. Accordingly, research that concerns specific behaviors and how they might be approached is regarded as useful for understanding and approaching other behaviors.

The purpose here is to provide suggestions regarding how to approach excessive behaviors in clinical practice. A variety of terms and definitions are used with respect to excessive behaviors, and this paper therefore begins with an overview of the terminology of excessive behaviors. Thereafter methods for identification and assessment, including questionnaires and interviews, as well as treatment needs, are presented. Qualitative researchers such as Gallegos (2005) and Honos-Webb and Leitner (2001) acknowledge that assessment and treatment occur in an interactional process between a unique client and a unique practitioner. Moreover, Southern (2007) emphasizes the importance of supervision for practitioners working with clients with difficulties with sexual behaviors, for example. Taken together, practitioners need to be attentive toward the client and strive to perceive what the client is communicating both explicitly and implicitly (Allen, 2008; Essig, 2012; Freimuth et al., 2008; Giugliano, 2006; Young, 2009). Processes that might hinder practitioners from approaching difficulties with excessive behaviors are reflected on toward the end of this paper.

It is not possible to present a complete overview of excessive behaviors, their identification, and their treatment. One reason is the varying terminology, which produces a vast number of possible combinations of terms when conducting searches on search engines such as PsycINFO and Google Scholar. Another reason is that there are a variety of approaches concerning when a behavior should be characterized as excessive, problematic, pathological, and in some cases as a disorder. The search strategy for this paper was to search for articles that concern a range of excessive behaviors. Because relatively few studies concern a range of excessive behaviors, articles that focused on one or two specific behaviors were also searched for and articles that concerned identification and treatment of excessive behaviors in mental health care were included. With these inclusion criteria, the number of articles exceeded what is possible to incorporate in a single paper. The articles that were found were evaluated with respect to their clinical significance as well as to the qualitative perspective of this specific journal. This means that prominence was given to articles with a focus on understanding excessive behaviors and articles that acknowledged the complexity of excessive behaviors and their treatment. The overview is thus incomplete and rather represents what the author evaluated as important to focus on in order to provide suggestions for how to approach excessive behaviors in mental health care.

## Terminology

The predominant terms that have been used with respect to excessive behaviors are *addiction*, *compulsion*, and *impulse control disorders* (Costorphine, Waller, Lawson, & Ganis, 2007; Fernandez-Aranda et al., 2006; Hartman et al., 2012; Mudry et al., 2011; Robbins & Clarke, 2015). The term *addiction* has become increasingly used and has more or less out-competed the other terms (Essig, 2012; Mudry et al., 2011; Robbins & Clark, 2015). However there is one exception, namely excessive sexual activities, for which the term *compulsivity* is often used, as in the journal named *Sexual Addiction & Compulsivity*.

Scholars of sociology direct attention toward how contemporary discourses influence how excessive behaviors are termed, categorized, and explained. Bailey (2005) and Elam (2015) describe how excessive behaviors as well as substance misuse are increasingly seen as representing an inherent medical disease that characterizes the individual concerned. Scholars working in disciplines that concern clinical practice also acknowledge the importance of terminology. Accordingly, it has been pointed out that the increasing use of the term *addiction* is connected to explanatory models that influence clinical practice, since the term is associated with a medicalization of human behaviors, as well as with essentialism, which means that behaviors are viewed as representing an inherent personal characteristic rather than a behavior that might be terminated or changed (Bailey, 2005; Essig, 2012; Schuckit, 2013; Sussman et al., 2011; Vrecko, 2010). The term moreover directs interest toward questions of tolerance, “loss of willpower,” and toward viewing excessive behaviors as an incurable disease, thereby neglecting underlying psychological and contextual factors, as well as alternative explanatory models (Giugliano, 2003; Karim & Chaudhri, 2012; Larkin, Wood, & Griffiths, 2006; Levine, 2010; Schuckit, 2013; Sussman et al., 2011). The view of excessive behaviors as a disease is influential. Thomas (2014) as well as Churucca, Perz, and Ussher (2014) describe how the disease model might contribute to a de-emphasis of agency, which in turn might lead to disbelief among practitioners that clients are able to change their life course. The term *addiction* also directs interest toward so-called rewards and the desirable nature of the behaviors, sometimes suggesting that the behaviors are repeatedly enacted because they, just like substances, provide desirable experiences that the individual repeatedly wants to achieve (Elam, 2015; Essig, 2012; Giugliano, 2006; Goodman, 2008; Grant, Potenza, Weinstein, & Gorelick, 2010; Karim & Chaudhri, 2012; Thomas, 2014). This view of excessive behaviors implies that the behaviors are experienced as rewarding and desirable, whereas in fact individuals who enact behaviors excessively might describe the behaviors as distasteful and enact them in order to confirm a negative and even degrading or shameful perception of themselves (Churucca et al., 2014; McKeague, 2014; Power, 2005; Punzi, Tidefors, & Fahlke, 2014). Shame has indeed been described as a core affect in suffering connected to negative self-perception, as well as a core affect in excessive behaviors and substance misuse (Nathanson, 1987; Wurmser, 1999). Substance misuse and excessive sexual behaviors might both be connected to feelings of shame that preceded the misuse and the excessive behaviors, and simultaneously the misuse as well as excessive behaviors might induce feelings of shame (Bromberg, 2001; Cook, 1988). Therefore, shame and self-perception need to be acknowledged in clinical practice concerning clients with excessive behaviors.

There are researchers who do not use terms that are connected to psychiatric classification systems, instead choosing terms such as *excessive* or *problematic behaviors* (Bonke & Borregaard, 2009; Caputo, 2015; Giugliano, 2003; Griffiths, Parke, & Wood, 2002; Orford, 2001; Thomas, 2014) and *misuse* or *overuse* (Essig, 2012; Mudry et al., 2011; Punzi, Tidefors, & Fahlke, 2015). In this paper, the term *excessive behaviors* is used, since it is not connected to any diagnostic category or to any specific explanatory model or theoretical approach. The term refers to a repetitive and paradoxical consummatory behavior that is enacted against one's “better judgment,” beyond what is experienced as tolerable and desirable (Benson & Eisenach, 2013; Churucca et al., 2014; Johnston et al., 2011; Larkin & Griffiths, 2002). Moreover, excessive behaviors are seen as desperate and repeated attempts to create solutions to suffering—attempts that fail but nevertheless are understandable and might hold the key to a more productive solution (Essig, 2012; Levine, 2010; Thomas, 2014).

## Identification and assessment

Considerable efforts have been made to develop questionnaires that identify specific excessive behaviors or a range of behaviors, and some of these questionnaires are said to provide a basis for a diagnosis. However it has been questioned whether the numerous questionnaires, and the variety of terms that are used in them, might obscure rather than enhance the capacity to understand excessive behaviors and the individuals who experience such difficulties (Griffiths et al., 2002; Levine, 2010). In the case of excessive sexual behaviors, there are for example questionnaires concerning sexual addiction, sexual dependency, perceived sexual control, hypersexual behavior, or sexual compulsivity (Hook, Hook, Davis, Worthington, & Penberthy, 2010). There are also questionnaires that assess compulsive Internet sex, Internet sex addiction, Internet addiction, smartphone addiction, online gaming addiction, or online gambling addiction (Hook et al., 2010; Robbins & Clark, 2015). Other questionnaires measure online shopping addiction, shopping addiction, compulsive shopping, compulsive buying, and compulsive spending, as well as tanning or exercise addiction, just to mention a few (Johnston et al., 2011; Pavarin & Biolcati, 2015; Sussman et al., 2011; Warner & Griffiths, 2006). The numerous terms and topics become unintentionally humoristic when they are all presented together. The purpose of this presentation is not to downgrade the efforts of the researchers who created these and other questionnaires. However, it seems sensible to ask whether increasing numbers of questionnaires with cut-off scores concerning criteria for a diagnosis are the primary needs of individuals who have difficulties with excessive behaviors. Some researchers argue that the use of questionnaires, cut-off scores, and diagnostic criteria supports acknowledgement of the difficulties concerned and thereby decreases the risk that excessive behaviors will pass unnoticed, and moreover that the capacity to provide appropriate treatment interventions is supported (Churucca et al., 2014; Grant et al., 2010; Hook et al., 2010; Karim & Chaudhri, 2012; Plant & Plant, 2003; Sussman et al., 2011).

Simultaneously, it has been argued that knowledge of excessive behaviors is more than a question of classification and questionnaires. In order to understand excessive behaviors, researchers as well as clinicians need to look beyond screening tools and acknowledge the client's life experiences (Caputo, 2015; Freimuth et al., 2008; Levine, 2010; Sussman et al., 2011). Practitioners in mental health care need to acknowledge the perceived meaning of the behaviors, as well as the life situation of the individual client, and it is beneficial to view the behavior as a form of nonverbal communication (Churucca et al., 2014; Essig, 2012; Young, 2009). Such an approach is essential when encountering clients whose excessive behaviors are attempts to handle traumatic experiences, neglect, and emotional, physical, and/or sexual abuse, since such experiences invoke overwhelming affects and thus are avoided by the individual concerned (Giugliano, 2006; Power, 2005; Young, 2009). The assessment process therefore needs to be flexible so that the practitioner is able to respectfully and patiently follow the concerns of the individual client, and it has to be acknowledged that such a process will take time (Power, 2005; Punzi et al., 2014; Thomas, 2014).

In research concerning substance abuse treatment, in which practitioners also encounter individuals who struggle to change their life course and behaviors, increased attention is paid to comprehensive assessment, encompassing the life circumstances of the client concerned (Keaney, 2006; Kellog & Tatarsky, 2012). Moreover, open-ended and flexible clinical interviews have the capacity to investigate topics that questionnaires do not detect and thereby flexible assessment processes have a therapeutic and motivational component (Keaney, 2006; Power, 2005; Thomas, 2014).

## Treatment needs

To the author's knowledge, no studies have investigated treatment concerning a range of excessive behaviors. With respect to the immense complexity of excessive behaviors, this lack of comprehensive studies is not surprising. Some researchers recommend psychoanalytic psychotherapy concerning, for example, excessive sexual activities (Giugliano, 2003, 2006) and computer use among teenagers and adults (Essig, 2012). By contrast, Young (2009) recommends family therapy when a teenager or young adult engages excessively in the Internet. Treasure, Claudino, and Zucker (2010) note that medication or cognitive behavioral therapy seem to be supportive for clients with difficulties with food intake. Altogether, the results from studies are somewhat scattered and indicate that no specific treatment of excessive behaviors can be said to be superior.

Researchers and clinicians nevertheless often ask what kind of treatment is most effective and what treatment should be recommended to clients with excessive behaviors. Perhaps the question should be posed from another perspective. It might be relevant to ask how practitioners in mental health care could approach excessive behaviors, how the treatment needs of the client concerned might be understood, and accordingly how an individualized treatment could be planned. Interestingly, researchers from various fields tend to advocate treatment in which a variety of approaches and interventions are integrated, especially for clients with severe difficulties and/or co-occurring difficulties (Freimuth et al., 2008; Iacovino, Gredysa, Altman, & Wilfley, 2012; McKeague, 2014; Power, 2005; Treasure et al., 2010). Instead of searching for the most efficient treatment and asking what methods practitioners should provide, it could be asked how practitioners could be empowered to use their capacity to identify and investigate excessive behaviors and to address them in an integrated treatment.

With such a perspective it becomes possible for mental health practitioners to plan an individualized treatment in which the whole life situation of the individual is acknowledged, rather than treating difficulties with excessive behaviors as isolated symptoms that might be removed. Moreover, it becomes possible to not only focus on the overt behaviors, but to support clients to tolerate, express, and handle overwhelming affects and to engage in intimate and social relationships, a need that researchers repeatedly point out as fundamental for a successful treatment (Caputo, 2015; Giugliano, 2006; Power, 2005; Reimersma & Sytsma, 2013; Young, 2009). Researchers have also argued that individual differences concerning etiology, gender, motivation, meaning, severity, and co-occurrence of other difficulties need to be addressed in research as well as in clinical treatment practice (McKeague, 2014; Parker & Guest, 2003; Robbins & Clark, 2015; Warner & Griffiths, 2006). For example, if the client is an adolescent who engages in online gaming to an excessive extent, it might be relevant to involve the family in the assessment process and in the treatment (Young, 2009). If the client, on the other hand, struggles with excessive behaviors that are connected to experiences of being sexually abused, it might be easier for the client to engage in a treatment in which family members and/or intimate partners are not involved (Giugliano, 2003, 2006; McKeague, 2014).

In summary, it seems reasonable that practitioners in mental health care seek to provide treatment that is based on clients’ needs and life situations, rather than on specific treatment interventions matched to certain diagnoses (Iacovino et al., 2012; Parker & Guest, 2003; Power, 2005; Young, 2009). It is also reasonable to pay attention to questions of gender differences and social norms when excessive behaviors are approached in clinical practice (McKeague, 2014; Plant et al., 2005; Giddens, 1995). For example, there is a higher occurrence of gambling, including excessive gambling, among men compared to women (Bonke & Borregaard, 2009; Svensson et al., 2011) and a higher occurrence of eating disorders among women compared to men (Nökleby, 2013; Treasure et al., 2010). Such differences need to be acknowledged, and simultaneously practitioners should not neglect the possibility that men might experience difficulties with food intake and women might experience difficulties with gambling. Another difference between men and women is that men have a higher occurrence of excessive sexual activities such as having multiple partners, engaging in excessive masturbation and use of pornography, and paying for sexual activities (Hook et al., 2010; Levine, 2010). However the predominant research on excessive sexual activities has concerned men, and thus there might be a bias toward traditionally male forms of sexual enactment. It should therefore be considered that women might experience difficulties with excessive sexual activities to the same extent as men, but their difficulties might be enacted in other ways than those who have traditionally been researched. For example, women might repeatedly engage in sexual activities on the conditions of others, might do so in a context that is controlled by men, and thereby might encounter considerable risk (Giddens, 1995; McKeague, 2014; Plant & Plant, 2003). The differences between men and women that have been mentioned here should not be seen as comprehensive or representative for each male or female client. Rather they illustrate the need to look beyond classification and diagnostic criteria and to acknowledge the perceived meaning, context, and life situation of the individual concerned.

## Don't forget the practitioners

While research studies tend to focus on general knowledge, practitioners in mental health care need to acknowledge the unique experience of each individual client, as well as general knowledge concerning identification, assessment,^1^ and treatment; practitioners’ capacity to make adequate clinical judgment should thus not be overlooked (Allen, 2008; Keen, 2012; Sharpless & Barber, 2009; Shedler, Mayman, & Manis, 1993). In order to make adequate clinical judgments concerning excessive behaviors, practitioners need to acknowledge the client's self-perception, life situation, subjective experiences, and co-occurring difficulties when treatment is planned (Griffiths et al., 2002; Parker & Guest, 2003; Power, 2005; Punzi et al., 2015). Moreover, practitioners need to make ethical considerations during assessment as well as during treatment (Allen, 2008; Sharpless & Barber, 2009). In this complex process, practitioners need to reflect on their own reactions, capacities, and shortcomings, as well as on their interaction with the client; otherwise adequate judgment and treatment might be hindered (Freimuth et al., 2008; Parker & Guest, 2003; Punzi et al., 2014; Southern, 2007).

The interaction between client and practitioner is expressed both verbally and nonverbally, and therefore the clinician must listen not only to what is being said, but also to what is not being said and what is being insinuated (Freimuth et al., 2008; Punzi et al., 2014; Shedler et al., 1993). There is a risk that practitioners may avoid sensitive topics such as difficulties with alcohol, sexuality, or food intake, both during assessment and treatment (Freimuth et al., 2008; Greenberg & Schoen, 2008; Shalev & Yerushalmi, 2009). Difficulties in acknowledging excessive behaviors are thus not necessarily connected to unawareness about the possibility that the client might experience excessive behaviors, but might also be connected to emotionally charged reactions and attitudes among practitioners (Dimen, 2005; MacDonald, 2008; Power, 2005; Southern, 2007). Therefore, it seems important to focus not only on the characteristics of the client. We need to focus on how we as practitioners might improve our capacity to have a dialogue about excessive behaviors and be prepared to listen to the considerable suffering in our client's experiences. We also have to find our own “blind spots.” If, for example, practitioners do not believe that sexuality is a relevant topic in treatment, they might be reluctant to raise questions about sexuality and may even be reluctant to deal with difficulties with sexuality that clients raise (Shalev & Yerushalmi, 2009). Another example is that difficulties with excessive shopping might be trivialized, and in such cases the capacity to identify and support clients with such difficulties will be considerably limited (Benson & Eisenach, 2013). Furthermore, difficulties with food intake are sometimes viewed as a female condition; this perception might decrease the capacity to identify difficulties with food intake among male clients, whereby treatment becomes insufficient (Cowan & Devine, 2008; Greenberg & Schoen, 2008; Nökleby, 2013).

It is sometimes argued that administration of questionnaires should become routine in mental health care since such routine would counteract the risk that excessive behaviors will pass unnoticed, but such an approach seems to be a simplification (Freimuth et al., 2008; Power, 2005; Punzi et al., 2014; Shedler et al., 1993). Questionnaires cannot distinguish answers that reflect genuine mental health from socially desirable answers, and moreover psychological defenses might influence the client to such an extent that he or she is unaware of difficulties and thus cannot report them (Shedler et al., 1993; Warner & Griffiths, 2006). Furthermore, it could be argued that over-occupation with technical mastery and structured methods might serve to disavow topics that are anxiety-provoking not only for clients but also for practitioners (Southern, 2007; Thomas, 2014). It also needs to be acknowledged that practitioners might react with explicit or implicit disgust, despair, or avoidance toward difficulties with excessive behaviors, and in such cases their reactions need to be acknowledged and worked through in a respectful manner, otherwise their capacity to support their clients is decreased (MacDonald, 2008; Power, 2005; Southern, 2007). Excessive behaviors are often self-destructive, and the self-inflicted negative consequences might awaken overwhelming reactions among the practitioners who encounter these clients. It is therefore important to encourage practitioners to respectfully raise questions about a range of excessive behaviors and to be prepared to listen to their clients’ experiences (Benson & Eisenach, 2013; Essig, 2012; Punzi & Fahlke, 2015). In order to support practitioners in listening to their clients’ suffering and handling their own reactions, it is recommended to provide adequate supervision in which excessive behaviors are not treated in trivializing or superficial ways (Shalev & Yerushalmi, 2009; Southern, 2007). Such an approach takes time, effort, and acknowledgment of the complexity of human life—phenomena that should be central to mental health care.

## Conclusions and future research

In summary, excessive behaviors are a phenomenon that has gained considerable attention among researchers. It is well known that excessive behaviors might co-occur with other difficulties and that they might accompany as well as replace each other. Most studies that have been performed are, however, based on questionnaires, and the knowledge achieved from such studies can neither reflect the lived experiences of individual clients, nor illustrate the complexities, ambiguities, and paradoxes that are important for understanding these behaviors. Researchers from various disciplines have pointed out that in treatment it is insufficient to focus on the overt behavior and its termination; Alma and Smaling (2006) emphasize that practitioners in mental health care need to use their capacity for empathic understanding and dialogue in clinical settings. According to Treasure et al. (2010), cognitive behavioral therapy for clients with difficulties with food intake needs to have a broader focus than thoughts and behaviors and to address interpersonal factors and self-esteem. Affective needs should be acknowledged among clients with difficulties with gambling (Caputo, 2015), food intake (MacDonald, 2008), and excessive sexual activities (Punzi et al., 2014). It has been recommended that treatment should not be seen as a structured intervention that should be matched to a specific behavioral diagnosis; instead treatment should be planned in accordance with the needs of the unique client. Thus, treatment ought to be based on individualized recommendations that seem relevant and adequate. Nevertheless, it is important to gain increased insight into how treatment needs might be understood and handled in mental health care and how treatment is planned in accordance with the needs of the unique client. Accordingly, future studies should investigate how clients as well as experienced practitioners perceive treatment needs and how an open and flexible approach, acknowledging the client's verbal as well as non-verbal communication, might enhance the possibility to plan an individualized treatment.

Excessive behaviors have negative consequences for the individual client as well as for relatives, partners, and friends, and increased understanding of ethical considerations, for example when clients continue to enact excessive behaviors despite negative consequences for themselves and/or others, is valuable. Ethical considerations also concern the question of how to define excessive behaviors, since a diagnosis of addiction, connected to the view of excessive behaviors as an inherent characteristic of the individual, might imply a perception of oneself as predestined to excessiveness, unable to change oneself and one's behaviors. The term *addiction* might also be connected to a disease model and as such used to explain, or even excuse, behaviors that are destructive for the individual and/or for others; in some cases the disease model diminishes acknowledgment of guilt and individual responsibility (Caputo, 2015; Churucca et al., 2014; Thomas, 2014). Individual responsibility should not be seen as an accusation, but rather as a human prerequisite that should be taken seriously. The question of responsibility is connected to the human capacity to grow and change one's life course, and discussions about perceived loss of control and responsibility might support individuals who struggle with excessive behaviors to emphasize agency and their capacity to terminate these behaviors. For example, Malterud and Ulriksen (2011) describe how clients who struggle with over-eating sense that practitioners might have a patronizing attitude toward them and how such an attitude fuels shame and the sense that personal responsibility is held against the client. Hence, future studies should focus on perceived agency and capacity to terminate excessive behaviors and on how diagnoses and explanatory models influence clients’ perceptions of their capacity to change.

Moreover, we need to understand how practitioners might be empowered to approach excessive behaviors and plan for an integrated treatment; we should specifically study how emotional reactions among practitioners support or decrease the possibility for identification and successful treatment. It seems important to gain increased understanding of the interaction between client and practitioner and of how practitioners might be supported to listen to their clients’ suffering. Therefore, studies should focus not only on the needs of the clients, but also on the needs of the practitioners who encounter the clients. Despite the considerable amount of research concerning excessive behaviors, the knowledge concerning reactions among practitioners and the importance of the clinical interaction, there are few if any studies that address these reactions and interactions specifically.

Qualitative studies have the capacity to contribute to knowledge about practitioners’ reactions and the interactional nature of treatment. Qualitative researchers could also contribute clinically important knowledge concerning the identification and assessment of excessive behaviors through attention to flexible assessment processes. Moreover, qualitative studies might contribute to conceptualizing the meaning of excessive behaviors, how individuals concerned perceive the etiology of these behaviors, and how behaviors can be excessively enacted in an attempt to solve emotional and relational difficulties (Caputo, 2015; Thomas, 2014; Young, 2009). Since the life situation of the individual and the context in which the behaviors are enacted need to be acknowledged, future studies should study how individuals of different age, gender, socioeconomic background, ethnicity, and/or sexual orientation perceive their behavior, themselves, and their lifeworld.

## Conflict of interest and funding

The author has not received any funding or benefits from industry or elsewhere to conduct this study.

## Note

1 In this paper, the term *assessment* refers to a process of understanding in which the client's narrative is central. The process might also involve structured assessment methods and various kinds of tests that are considered beneficial for understanding the client concerned.
